# Mimicking osteoid 3D porous dense microfiber silk fibroin embedded poly(vinyl alcohol) scaffold for alveolar ridge preservation

**DOI:** 10.1093/rb/rbae130

**Published:** 2024-11-13

**Authors:** Supaporn Sangkert, Perumal Ramesh Kannan, Jirut Meesane, Kanokporn Santavalimp, Jutharat Phongthanawarakun, Walaiporn Promkaew, Wachiratan Anupan, Nuttawut Thuaksuban

**Affiliations:** Department of Oral and Maxillofacial Surgery, Faculty of Dentistry, Prince of Songkla University, Hatyai 90110, Thailand; Institute of Smart Biomedical Materials, School of Materials Science and Engineering, Zhejiang Sci-Tech University, Hangzhou 310018, China; Zhejiang-Mauritius Joint Research Center for Biomaterials and Tissue Engineering, Zhejiang Sci-TechUniversity, Hangzhou 310018, China; Division of Biomedical Science and Biomedical Engineering, Faculty of Medicine, Prince of Songkla University, Hatyai 90110, Thailand; Department of Oral and Maxillofacial Surgery, Faculty of Dentistry, Prince of Songkla University, Hatyai 90110, Thailand; Department of Oral and Maxillofacial Surgery, Faculty of Dentistry, Prince of Songkla University, Hatyai 90110, Thailand; Department of Oral and Maxillofacial Surgery, Faculty of Dentistry, Prince of Songkla University, Hatyai 90110, Thailand; Department of Oral Diagnostic Sciences, Faculty of Dentistry, Prince of Songkla University, Songkhla 90110, Thailand; Department of Oral and Maxillofacial Surgery, Faculty of Dentistry, Prince of Songkla University, Hatyai 90110, Thailand

**Keywords:** mimicking, osteoid, scaffold, microfiber, silk fibroin, poly(vinyl alcohol), alveolar ridge preservation

## Abstract

Alveolar ridge loss presents difficulties for implant placement and stability. To address this, alveolar ridge preservation (ARP) is required to maintain bone and avoid the need for ridge augmentation using socket grafting. In this study, a scaffold for ARP was created by fabricating a 3D porous dense microfiber silk fibroin (mSF) embedded in poly(vinyl alcohol) (PVA), which mimics the osteoid template. The research utilized a freeze–thawing technique to create a mimicked osteoid 3D porous scaffold by incorporating different amounts of mSF into the PVA, namely, 1%, 3%, 5% and 7%. Subsequently, a 3D profilometer machine and a scanning electron microscope were employed to examine the morphology and size of the mSF and the mimicked osteoid 3D porous scaffold in all groups. Thermal characteristics and crystalline structure were analyzed before assessing the water contact angle, swelling behavior, degradation and mechanical properties. The experiment evaluated the biological performance of the mimicked osteoid 3D porous scaffold by examining the efficacy of osteoblast cell adhesion, proliferation, viability, protein synthesis, alkaline phosphatase (ALP) activity and calcium synthesis. Finally, the ability of osteoblast cells to regulate the osteoid matrix deposition on the osteoid 3D porous scaffold was assessed by mimicking the dynamic bone environment using rat mesenchymal stem cells. The findings suggest that incorporating mSF into PVA enhances the interconnective pore size, crystalline structure and thermal behavior of the mimicked osteoid 3D porous scaffold. The hydrophilicity of PVA decreased with an increase in the proportion of mSF, while a higher proportion of mSF resulted in increased swelling and mechanical characteristics. Incorporating a greater proportion of mSF, specifically 5% and 7%, led to a reduced rate of degradation. The addition of 5% mSF to the PVA 3D porous scaffold resulted in remarkable biological properties and excellent osteoconductive activity.

## Graphical Abstract

**Figure:**
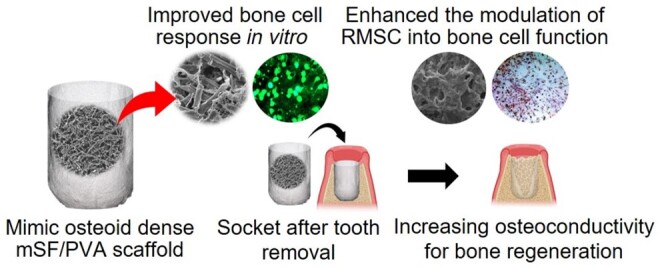

## Introduction

It is well established that insufficient bone volume compromises implant stability and long-term success. Resorption of bone after tooth loss leads to alveolar ridge loss, a nearly unavoidable issue that decreases both the vertical and horizontal dimensions of the ridge [1]. This loss has a significant impact on the success rate of dental implant procedures because there is inadequate bone volume for optimal implant placement [2]. Despite this, bone augmentation remains necessary when the bone volume is insufficient to support the implant. Numerous studies indicate that the rate of bone loss following tooth extraction is initially high within the first 6 months, subsequently increasing by approximately 0.5–1% annually [3]. Currently, a method for alveolar ridge preservation (ARP) involves preserving the alveolar bone and eliminating the necessity for ridge augmentation through socket grafting [4]. Three primary types of socket scaffold materials exist: autograft, allograft and xenograft. However, these methods present specific difficulties, such as the potential transmission of diseases, immunological rejection, and restrictions on donor sizes [5, 6]. An alternate method for regenerating new bone involves implanting socket scaffolds based on tissue engineering principles, specifically by mimicking natural structures. This approach has the advantage of preserving the defect space and promoting bone restoration [7, 8].

Mimicking represents a fascinating approach to enhancing the biological functionality of scaffolds [9, 10]. The replication of the bone extracellular matrix (ECM) structure has been shown to improve the response of osteoblast cells to the scaffold, thereby enhancing the construction of the osteoid template for bone formation. These improvements include cell proliferation, cell migration and calcification during the differentiation process [11, 12]. Several studies demonstrate the crucial role of protein fiber in regulating the osteoid template, as well as the activation of signaling pathways for bone regeneration during the bone healing process [11, 13]. Furthermore, the fiber-like network structure of protein provides an elastic network capable of withstanding tension and aiding in mineral deposition and resorption processes, which are essential for new bone tissue formation [14, 15]. Therefore, mimicking the fibrous protein structures encountered close to the ECM can enhance the strength and biological characteristics of the scaffold [16, 17].

Poly(vinyl alcohol) (PVA), a biocompatible synthetic polymer, has been widely used as a scaffold for bone tissue generation due to its significant advantages in the structure of 3D porous scaffolds [18, 19]. The PVA porous scaffold facilitates cell migration and promotes the development of new tissue with simplicity [20]. The hydrophilic properties of PVA support nutrition flow and protein absorption, which assist in cell attachment [21, 22]. Furthermore, the adhesive property of PVA is crucial for attachment to the bone ECM [23]. PVA was selected to mimic the binding ECM and provide a ground substrate for protein fiber immersion in the formation of the osteoid bone template.

SF is a protein polymer composed of three primary amino acids: glycine, alanine and serine, each present in different amounts [24]. SF is characterized by the presence of bioactive compounds, which are consistently employed in combination with other materials to enhance the biological functionality of scaffolds [25, 26]. Several studies indicate that the SF microfiber can enhance cell adhesion, migration and proliferation [27]. Furthermore, SF microfibers play a crucial role in the process of calcium deposition during calcification [28], functioning as a protein within the ECM protein network in bone [29]. This research utilized SF microfibers to mimic the fibrous protein network in the bone ECM and stimulate the osteoid bone template in the porous scaffold.

**Figure 1.**
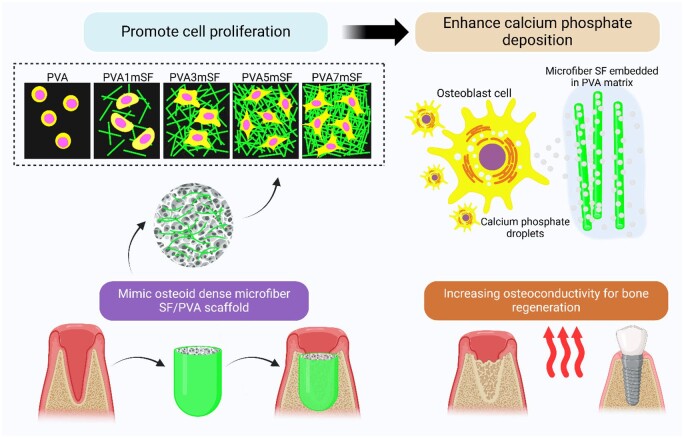
Schematic illustration of the mimicked osteoid 3D porous scaffold for alveolar ridge preservation.

This research aims to develop a mimicked osteoid 3D porous scaffold by utilizing PVA as the base material and SF microfibers to imitate the fibrous protein structure. These fibers are immersed in the PVA base and serve as an ECM structure with the ability to generate the osteoid bone template as shown in Figure 1. This investigation assessed the appearance, morphological characteristics, biological properties and calcification performance of the mimicked osteoid 3D porous scaffold, as well as its capacity to stimulate the transformation of rat mesenchymal stem cell (RMSC) into bone cells in mimicked bone environment with a dynamic condition model.

## Materials and methods

### Silk fibroin fiber preparation

After the degumming procedure, the SF fiber was dried in a hot air oven at 70°C for 4 hours. The preparation of SF microfiber involved the following steps: 4.2 g of SF fiber were combined with 21 g of sodium hydroxide, and then 60 ml of dH_2_O was added to initiate an alkaline hydrolysis action. After 60 minutes, an additional 60 ml of boiling dH_2_O was introduced to continue the reaction. The SF microfiber was then rinsed with dH_2_O multiple times using the centrifuge technique. The pH of the microfiber was neutralized with a solution of HCl/NaOH. Finally, the SF microfiber underwent freeze-drying for 24 hours and was subsequently stored in a desiccator until use [28].

### PVA-microfiber silk fibroin osteoid 3D porous scaffold preparation

Three percent (3%) of PVA was dissolved in dH_2_O at 80°C for 30 minutes, with continuous stirring during the process. Various concentrations of SF microfibers were mixed with the PVA solution at concentrations of 1%, 3%, 5% and 7% and stirred for 30 minutes. The PVA/SF microfiber solutions were then poured into the hydrophobic plastic block for each group. A hydrogel network was formed by subjecting the mixtures to five cycles of freeze–thawing, where the solutions were kept at −80 °C for 4 hours and then placed at room temperature for 4 hours. Following the completion of freeze−thawing cycles, the PVA/SF microfibers were freeze-dried for 24 hours, resulting in the formation of a porous sponge structure for further tests.

### Characterizations

The morphology, surface structure and pore size distribution of the mimic osteoid 3D porous scaffold composed of microfiber silk fibroin (mSF) were investigated using a 3D profilometer machine (Keyence VR-6200), an inverted microscope (Axio Observer, Carl Zeiss) and scanning electron microscope (SEM) (Quanta 400, FEI, Brno, Czech Republic). For SEM observation, the samples were coated with gold using a gold sputter coating machine. The length and thickness of mSF, as well as the pore size of the mimic osteoid 3D porous scaffold, were calculated using the ImageJ tool. SEM images were randomly selected from three regions for analysis, with a total of 25 measurements taken. The crystalline structure of the samples in each group was analyzed using an X-ray diffraction (XRD) machine (X’Pert MPD; Philips, The Netherlands). Samples from each group were placed into the XRD instrument, and diffraction patterns were scanned from 5θ to 90θ, with a scanning step set at 0.05θ per second. The thermal characteristics of the mimic osteoid 3D porous scaffold, particularly its heat absorption and release abilities, were evaluated using a DSC machine (DSC7, Perkin Elmer, USA). Before the tests, the samples were stored in a desiccator for 24 hours. Subsequently, the samples were cut into small pieces, and their weights were obtained before being placed in an aluminum pan. The temperature was controlled and set within the range of 25°C to 350°C.

### Water contact angle

Before the freeze-drying procedure, 100 µl of the sample solution from each group was placed onto a glass slide to create a thin layer of film and subjected to five cycles of freeze–thawing. Then, 3 µl of dH_2_O was dropped onto a random region of the thin film. The angle was determined using a water contact angle machine (Optical Contact Angle Analyzer, OCA25; Data Physics Instruments GmbH, Filderstadt, Germany).

### Physical properties testing

The swelling properties of the mimic osteoid 3D porous scaffolds were individually evaluated by recording the sample weight before and after the testing process. Each sample, with a diameter of 10 mm, was soaked in PBS at 37°C for varying time points, including 0.5, 1, 2, 4, 8, 12 and 24 hours. The swelling ratio was determined using the following formula, where *Ws* and *Wd* represent the weight of the swollen and dry samples, respectively.

$$\mathrm{Swelling}\;\mathrm{ratio}\;(\%)\;=\frac{\;Ws-Wd}{Wd}\;\times \;100$$

The degradation was assessed by immersing each sample in PBS at 37°C for different times, including 3, 7, 14, 21 and 27 days. After the desired time intervals, the PBS solution was removed, and the samples were rinsed multiple times with dH_2_O before being stored at −80°C for 24 hours and subjected to freeze-drying. The degradation calculation was performed using the following formula:

$$\mathrm{Degradation}\;\mathrm{efficiency}\;=\;\frac{Wi-Wf}{Wi}\times 100$$

*Wi* represents the initial weight of the membrane, while *Wf* represents the weight of the sample at various time intervals.

The mechanical properties of the mimic osteoid 3D porous bone plugs were assessed using universal testing equipment (Lloyd model LRX-Plus, Lloyd Instrument Ltd, London, UK). The evaluation included measuring maximum loading, Young’s modulus and stiffness. Before testing, all samples were trimmed to a diameter of 10 mm and a thickness of 5 mm. The compression testing was conducted at a velocity of 0.5 mm/min, with the parameter set at 250 N. A 40% strain threshold was established to terminate the testing process. Young’s modulus was determined by analyzing the linear portion of the compressive stress-strain curve, whereas stiffness was obtained by measuring the resistance to elastic deformation when a load was applied.

### Protein adsorption

The protein adsorption was evaluated at 4 and 24 hours by the following steps: all samples were cut with 8 mm in diameter, immersed in a complete DMEM medium containing 10% FBS, and incubated for 4 and 24 hours. All samples were washed twice with PBS for protein residual removal and dried for around 1 hour at 37°C. The protein adsorption on the sample was eluted by using a 1% sodium dodecyl sulfate solution for 30 minutes before being measured by the BCA assay kit.

### Cell culture

The study was approved by the ethical committee under approval number NH6708-011. The MC3T3-E1 cell line was cultured in alpha-MEM medium (α-MEM; Gibco^®^, Invitrogen^TM^, Carlsbad, CA) supplemented with 10% fetal bovine serum, 1% penicillin/streptomycin, and 0.1% fungizone. For the differentiation step, the completed α-MEM was further supplemented with 100 nM dexamethasone, 50 µM ascorbic acid and 20 mM β-glycerophosphate to produce the osteogenic medium.

### Cell adhesion efficacy after 4 hours of cell seeding

The cell adhesion efficacy of the mimic osteoid 3D porous scaffold was tested by washing the material several times with PBS after 4 hours of cell seeding using PrestoBlue^®^ (PrestoBlue^®^ Cell Viability Reagent, Invitrogen). A solution was prepared by combining 10% PrestoBlue^®^ in the completed α-MEM, and 300 µl of this solution was added to each sample before incubation at 37°C. The mixture was then evaluated for viability. Following the incubation period, 200 µl from each group was transferred to a 96-well plate, and the resulting concentration was determined at a wavelength of 600 nm.

### Cell proliferation

To assess the biocompatibility of the mimic osteoid 3D porous scaffold, the MC3T3-E1 cells were seeded onto the surface of the samples at a density of approximately 100 000 cells per sample. The cell proliferation was assessed on days 1, 3, 5 and 7, with media being changed on alternative days.

### Cell viability

The prepared scaffold material was tested for the viability of MC3T3-E1 cells on day 1. After treatment, the media was removed, and the cells were washed with PBS twice. Then, 1 ml of complete α-MEM containing 5 µl of 5 mg/ml fluorescein diacetate (FDA) in acetone was added. The mixture was incubated at 37°C for 5 minutes. After removing the solution, the sample was rinsed with PBS several times. The live cells were then examined using a fluorescence microscope.

### Total protein assay

The evaluation of protein synthesis and the deposition of the protein matrix of osteoblast cells on the mimic osteoid 3D porous scaffold were performed on days 7, 14 and 21. The samples in 1% Triton-X 100 in PBS were subjected to a freeze–thaw cycle of −80°C for 4 hours followed by room temperature for 4 hours, repeated three times. After the freeze–thaw cycles, the samples were pulverized, and the solution was collected. The supernatant (50 µl) was obtained after centrifugation and used for BCA testing (Thermo Scientific, USA). Protein quantification was performed on the solutions according to the manufacturer’s guidelines.

### Alkaline phosphatase activity

Alkaline phosphatase (ALP) activity was assessed on days 7, 14 and 21. After two washes with PBS, each sample was treated with 800 µl of lysis buffer containing 1% Triton-X in PBS. Three freeze–thaw cycles were employed to induce cell membrane disruption, followed by centrifugation to collect the supernatant. The ALP activity was quantified from the supernatant using an ALP assay kit obtained from Abcam (Cambridge, UK).

### Calcium synthesis

On days 7, 14 and 21, all groups were rinsed twice with PBS, and 800 µl of lysis buffer was added to each sample. The addition of lysis buffer caused cell membrane disruption through a freeze–thaw procedure, repeated for three cycles. Following centrifugation, the debris was removed, and the resulting solution was used for a calcium colorimetric assay kit (Sigma-Aldrich).

### Alizarin red staining

Calcium nodule deposition on the membrane was observed after days 7 and 14 using Alizarin red staining. The membranes in each group were washed twice with PBS, followed by fixation with 4% formalin for 15 minutes at room temperature. The samples were then washed several times with dH_2_O to remove unbound Alizarin red. The calcium nodules were observed using microscopy.

### Mimicking bone environment with dynamic condition

On day 21 of the differentiation stage, the seeded mimic osteoid 3D porous scaffold was removed from the osteoblast cell. The scaffold was then used to create scaffolds made of decellularized ECM (dECM) employing trypsin-EDTA (1X) and sodium dodecyl sulfate (SDS)-based decellularization techniques. The dECM mimic osteoid 3D porous scaffold, which had been seeded with osteoblast cells, underwent two washes with PBS before being immersed in a 0.25% Trypsin-EDTA solution for 24 hours at 37°C. This was followed by several washes with PBS solution. Subsequently, the sample was treated with a 0.1% SDS solution for 6 hours at 37 °C with agitation and thoroughly washed with PBS [30]. Afterward, it was stored at −80°C overnight and then freeze-dried to obtain the dECM osteoid 3D porous scaffold for further analysis.

The efficacy of the dECM mimic osteoid 3D porous scaffold in promoting biofunctionality and cellular development was assessed using rat mesenchymal stem cells (RMSC, Cell Application, Inc.). The RMSC cells were initially seeded in the dECM mimic osteoid 3D porous scaffold at a density of 2 × 10^4^ cells under dynamic conditions, with the incubate shaker set at 100 rpm. The proliferation of the RMSC was then assessed on days 1, 3, 5 and 7 using the PrestoBlue^®^ reagent. Cellular adhesion was examined using SEM on day 14 according to the following procedure: the dECM mimic osteoid 3D porous scaffold was rinsed twice with PBS and then fixed with 4% formaldehyde for 15 minutes at room temperature. Before examination using the SEM machine, the dECM mimic osteoid 3D porous scaffold was dried out by freeze-drying. The efficacy of the dECM mimic osteoid 3D porous scaffold in enhancing RMSC and stimulating bone cell function was assessed on days 14 and 21 using ALP activity, alizarin red staining, alizarin red quantitative analysis and OCN measurements. The OCN measurement was performed by testing the collected solution from the cell lysis procedure in all groups using the Mouse Osteocalcin Enzyme Immunoassay Kit, BT-470 (Alfa Aesar, USA), with a volume of 25 µl for each sample. The procedure was conducted in accordance with the guidelines provided by the manufacturer.

### Statistical analysis

The statistical data were analyzed using a one-way analysis of variance and Tukey’s honesty significant difference test. The data were collected from the sample groups (*n* = 5) and are displayed as the mean ± standard deviation (SD).Statistical significance was defined as *P < 0.05, and **P < 0.01.

## Results

### The morphology of mSF

**Figure 2.**
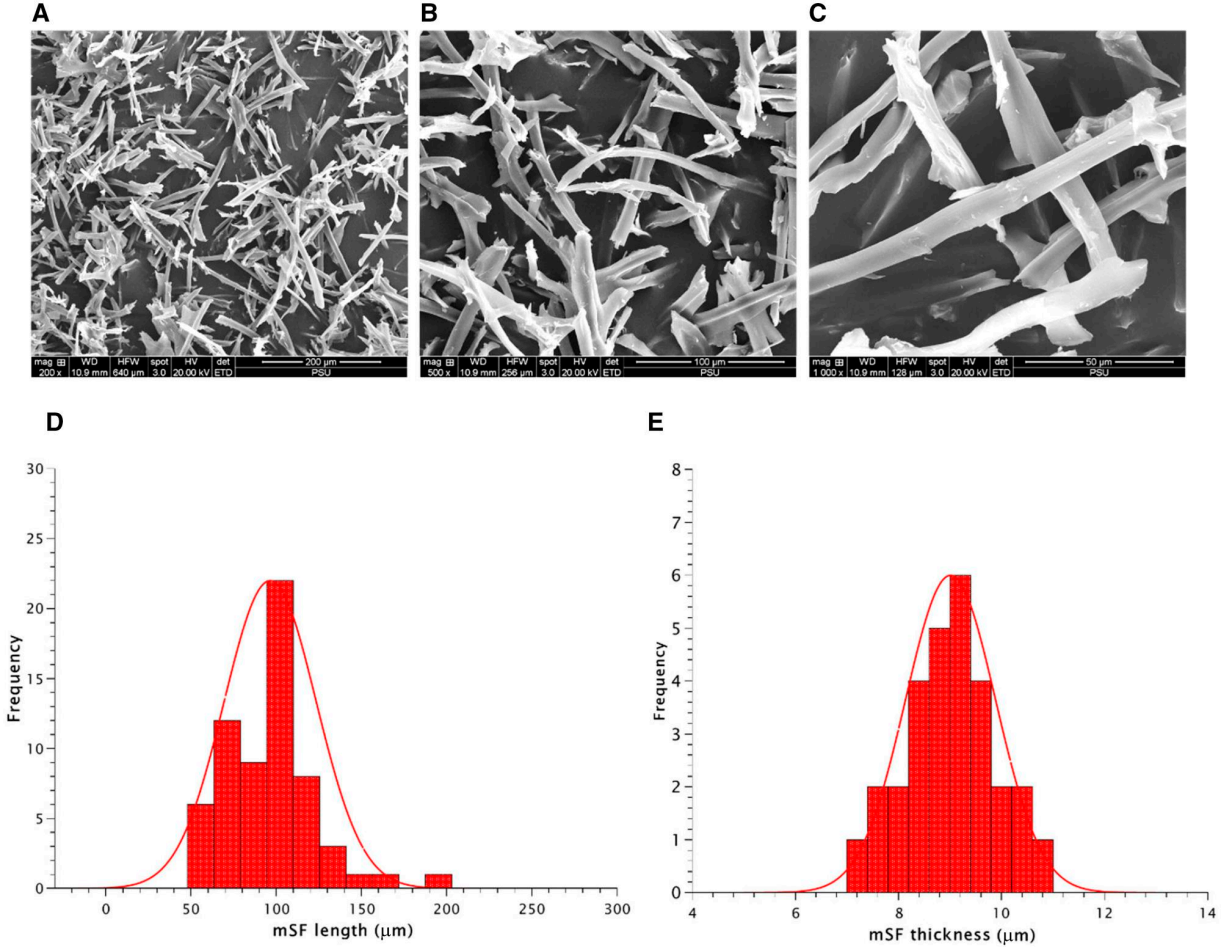
The morphological structure of the mimic osteoid 3D porous scaffold in each group after observed using an SEM machine.

The morphology of mSF displayed uniform length and thickness, as well as a smooth surface (Figure 2A–C). The average length of mSF was approximately 102.25 ± 27.07 µm (Figure 2D), while the average thickness was 9.01 ± 0.87 µm (Figure 2E).

### The morphological structure of PVA/mSF 3D porous scaffold

**Figure 3.**
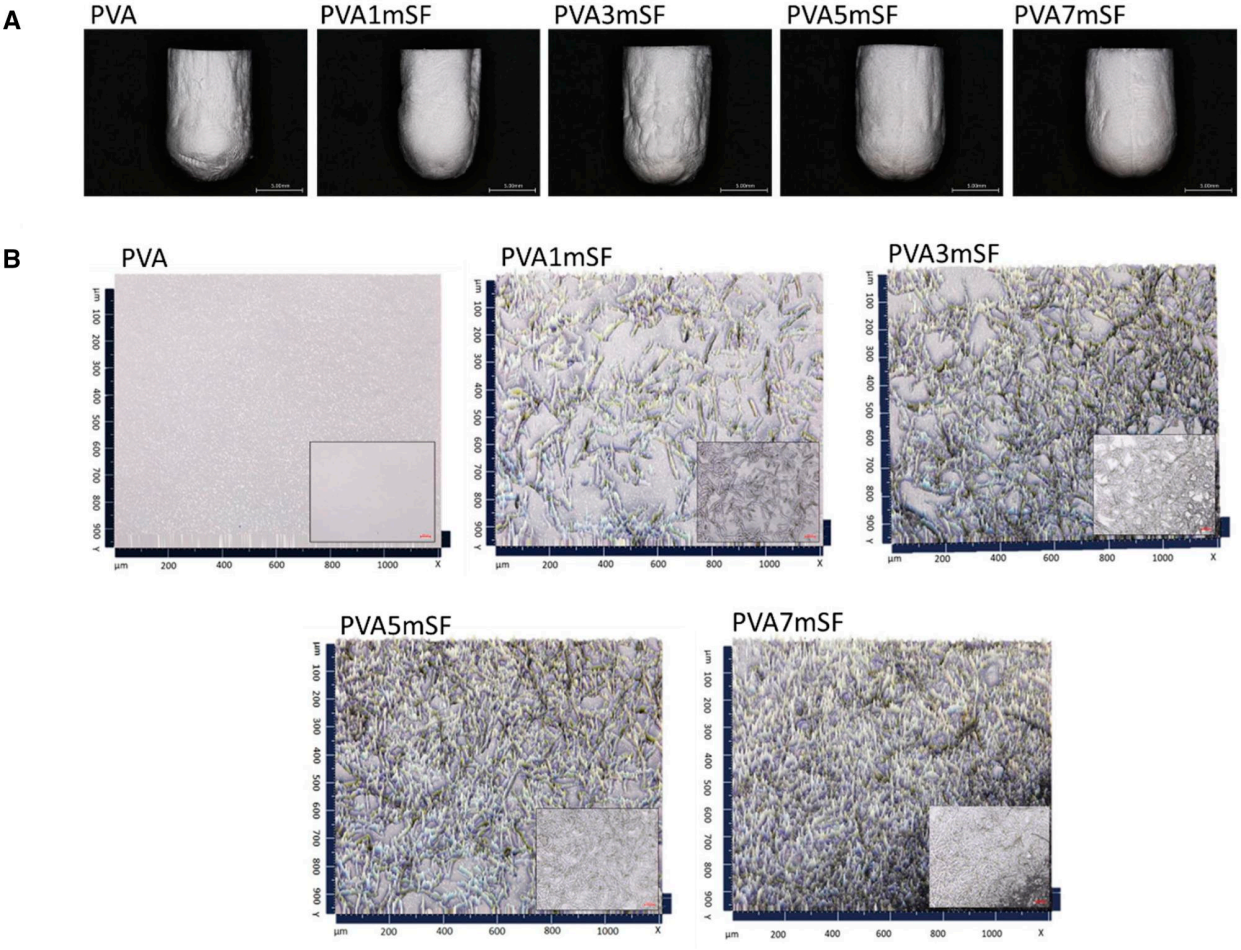
The mimic osteoid 3D porous scaffold in each group (**A**) and the mSF embedded in the PVA (**B**).

All 3D porous scaffold groups that mimic osteoid appeared white (Figure 3A). The modified PVA with mSF groups showed increased roughness, particularly at higher mSF percentages, resulting in a rough surface morphology. Additionally, a dense mSF network structure was observed with increasing mSF concentration in PVA (Figure 3B).

### Morphological structure

**Figure 4.**
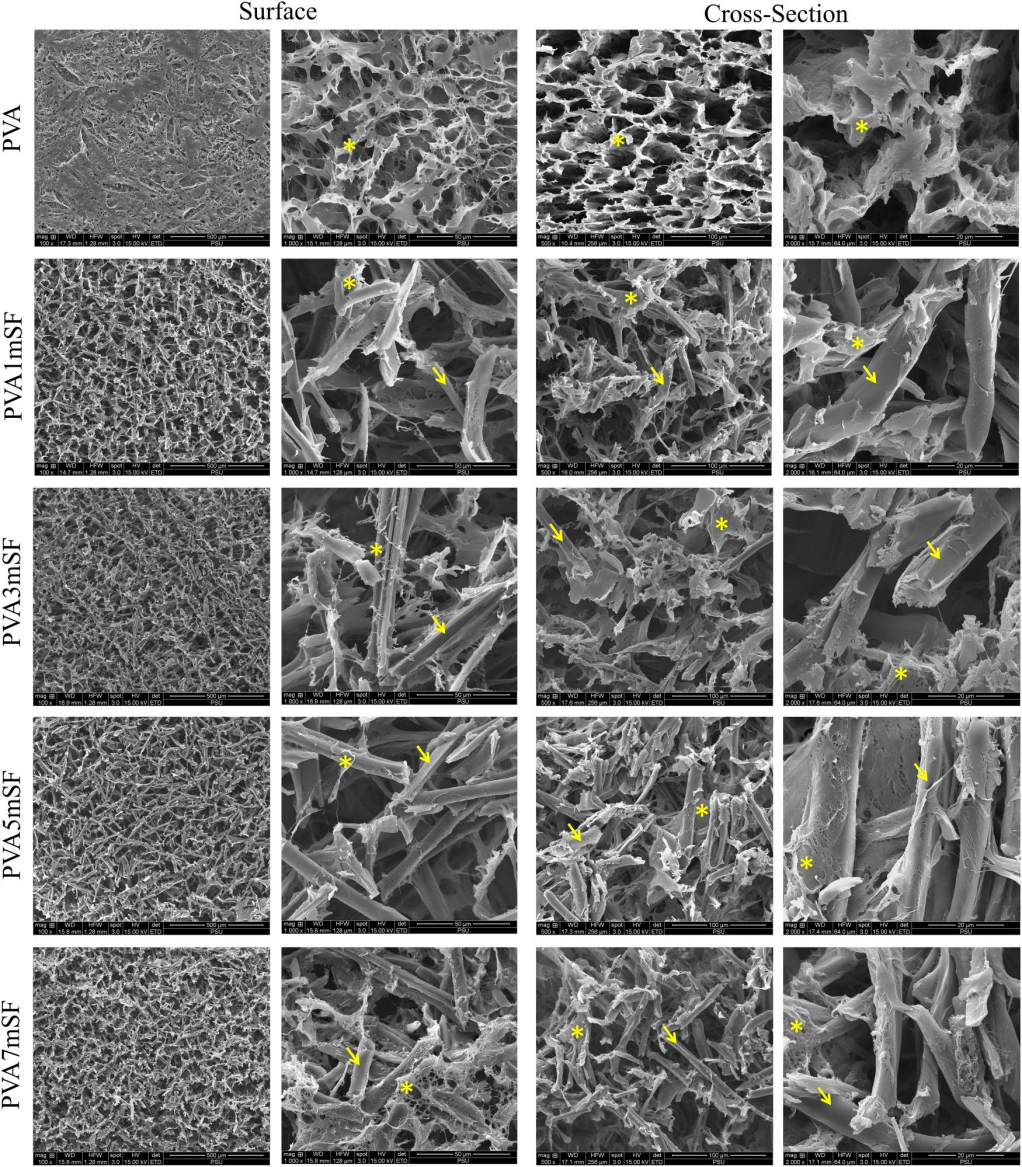
The structural morphology of the mimic osteoid 3D porous scaffold in each group was observed using an SEM machine. The arrows indicate the mSF, whereas the asterisks (*) point to the PVA.

The findings indicated a substantial difference between the group that used a combination of PVA and mSF and the group that utilized mainly pure PVA (Figure 4). The presence of mSF (arrows) in the PVA (asterisks) enhanced the porous structure of the mimic osteoid 3D porous scaffold. This enhancement was influenced by the percentage of mSF, with a greater percentage resulting in smaller pore sizes. The order of increasing effect on pore size was as follows: PVA, PVA1mSF, PVA3mSF, PVA5mSF and PVA7mSF. Additionally, a higher percentage of mSF favored the dense structure.

### XRD and differential scanning calorimetry patterns

**Figure 5.**
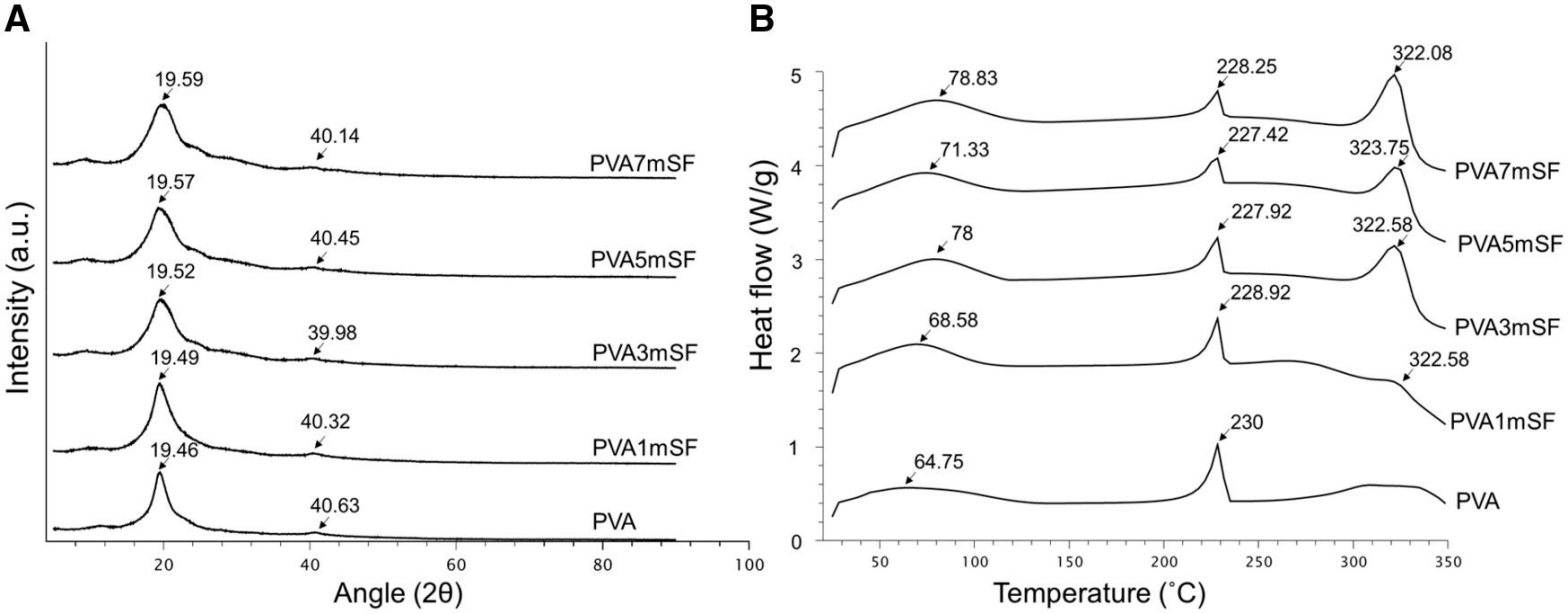
XRD analysis of the crystalline structure (**A**) and DSC thermogram of the mimic osteoid 3D porous scaffold (**B**).

The pure PVA group exhibited a distinct peak at 2θ = 19.46° and a small shoulder peak at around 40.63°, indicating the characteristic peaks of the PVA crystalline structure. Different concentrations of mSF in PVA, including 1mSF, 3mSF, 5mSF and 7mSF, showed similar XRD patterns with a slight shift in the crystalline structure. The small shoulder peak of PVA decreased when combined with mSF (Figure 5A).

The DSC machine was used to assess the thermal behavior of the mimic osteoid 3D porous scaffold. The first peak observed indicated the endothermic glass transition (*Tg*), or melting point, resulting from the water evaporation within the structure. The PVA group had a lower melting temperature of approximately 64.75°C compared to the other groups. The melting temperature of the mimic osteoid 3D porous scaffold increased when combined with mSF, particularly as the proportion of mSF increased (1mSF, 3mSF, 5mSF and 7mSF). The second exothermic peak represents the movement of molecules within the α-crystal structure when heated. An intense endothermic peak was detected at around 230°C in the PVA mimic osteoid 3D porous scaffold, slightly above the temperatures observed in PVA1mSF, PVA7mSF, PVA3mSF and PVA5mSF. The third peak was identified as an endothermic peak resulting from the thermal decomposition of mSF in PVA following modification. The activation temperatures required to break the structures of 1mSF, 3mSF, 5mSF and 7mSF in PVA were 322.58, 322.58, 323.75 and 322.08°C, respectively (Figure 5B).

### Water contact angle

**Figure 6.**
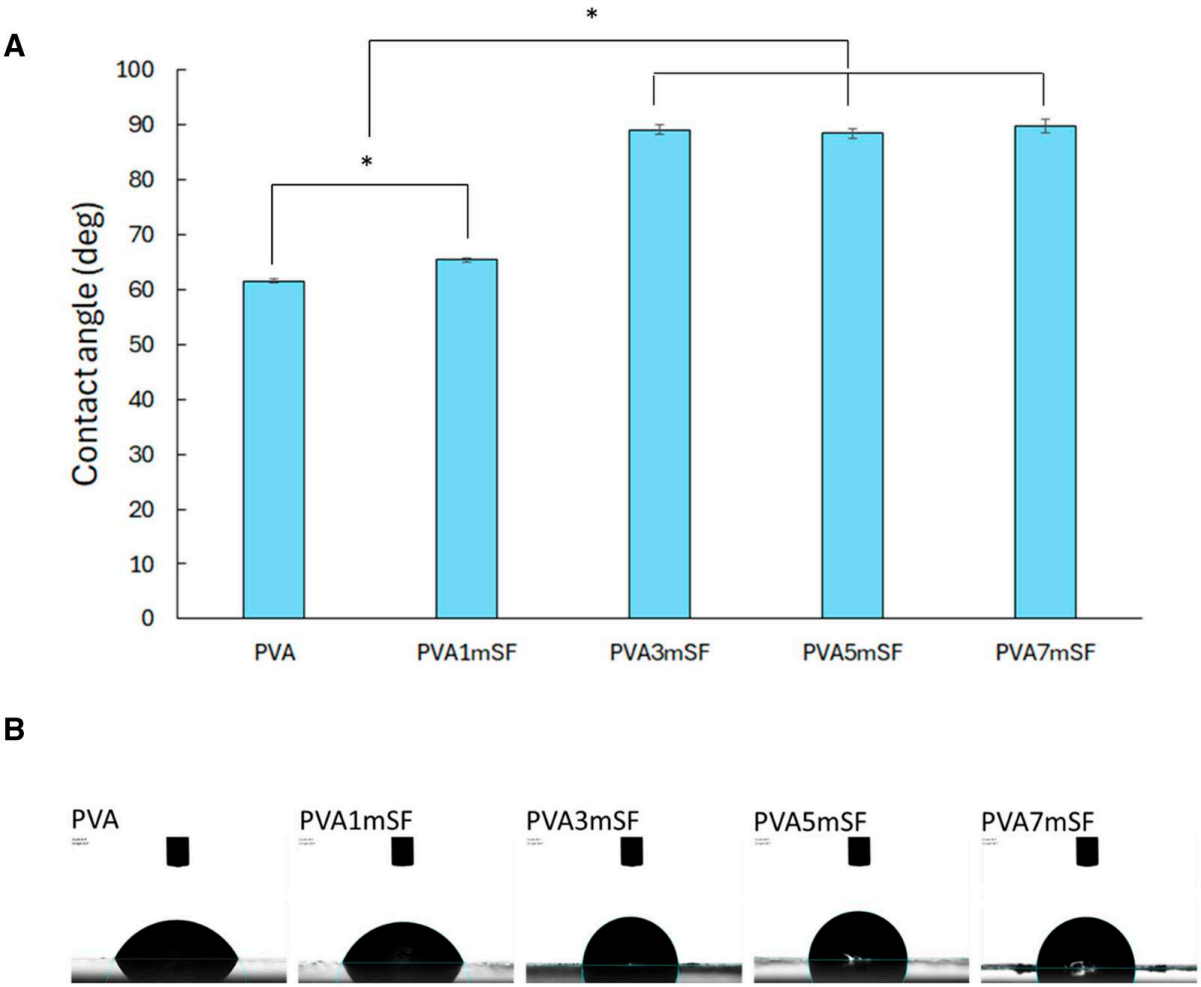
Water contact angle of the mimic osteoid 3D porous scaffold in each group (**A**), along with representative images of water droplets on the scaffold surface (**B**).

The results indicate that the pure PVA group exhibited superior hydrophilicity compared to PVA1mSF, particularly in the groups with a higher proportion of mSF, namely PVA3mSF, PVA5mSF and PVA7mSF. The contact angle was larger in PVA3mSF, PVA7mSF and PVA5mSF, indicating lower hydrophilic characteristics (Figure 6A and B). There were no significant differences observed among these groups.

### Physical properties

**Figure 7.**
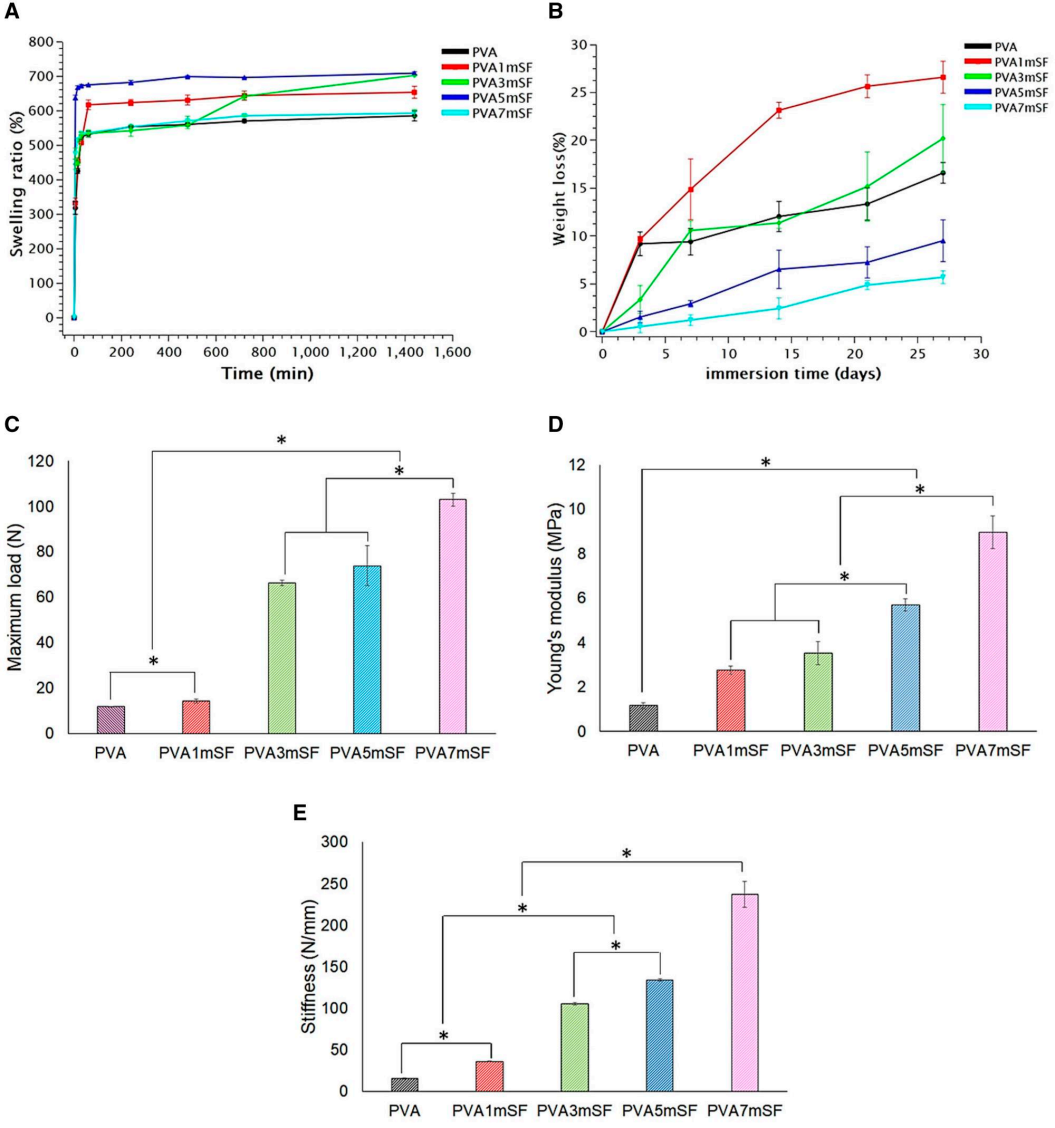
Swelling property of the mimic osteoid 3D porous scaffold (**A**), along with its weight loss (**B**), maximum load (**C**), young’s modulus (**D**), and stiffness (**E**).

The swelling properties of the mimic osteoid 3D porous scaffold were assessed in each group over a period ranging from 5 minutes to 1440. The findings indicated a significant increase in the swelling ratio within the initial 5–15 minutes across all groups (Figure 7A).

The pure PVA group exhibited greater levels of swelling compared to the other groups at all measured time intervals. The swelling properties of the mimic osteoid 3D porous scaffold were influenced by the amount of microfiber SF, with an increase in mSF resulting in a decrease in the swelling ratio. Furthermore, both PVA5mSF and PVA7mSF exhibited decreased swelling values compared to PVA3mSF, PVA1mSF and PVA groups. The degradation of the mimic osteoid 3D porous scaffold was evaluated on days 3, 7, 14, 21 and 27 (Figure 7B). The findings indicated that the degree of degradation was significantly greater in the PVA and PVA1mSF groups compared to the PVA3mSF, PVA5mSF and PVA7mSF groups during the initial phase. The rate of degradation increased over time. PVA1mSF showed a higher degree of degradation compared to the other groups at all time points until day 27. The degradation behavior of PVA and PVA3mSF was similar. The degradation rate of the PVA5mSF and PVA7mSF groups was lower. The mechanical characteristics of the PVA group were enhanced following the incorporation of mSF, increasing Young’s modulus (Figure 7D). Statistically significant differences were observed between each group, with PVA7mSF exhibiting the greatest values compared to PVA5mSF, PVA3mSF, PVA1mSF and PVA, respectively. The maximum load and stiffness data showed a similar trend to Young’s modulus, indicating an improvement of PVA with mSF (Figure 7C). This improvement was particularly notable in the PVA7mSF group, which had the highest proportion of mSF.

### Protein adsorption

**Figure 8.**
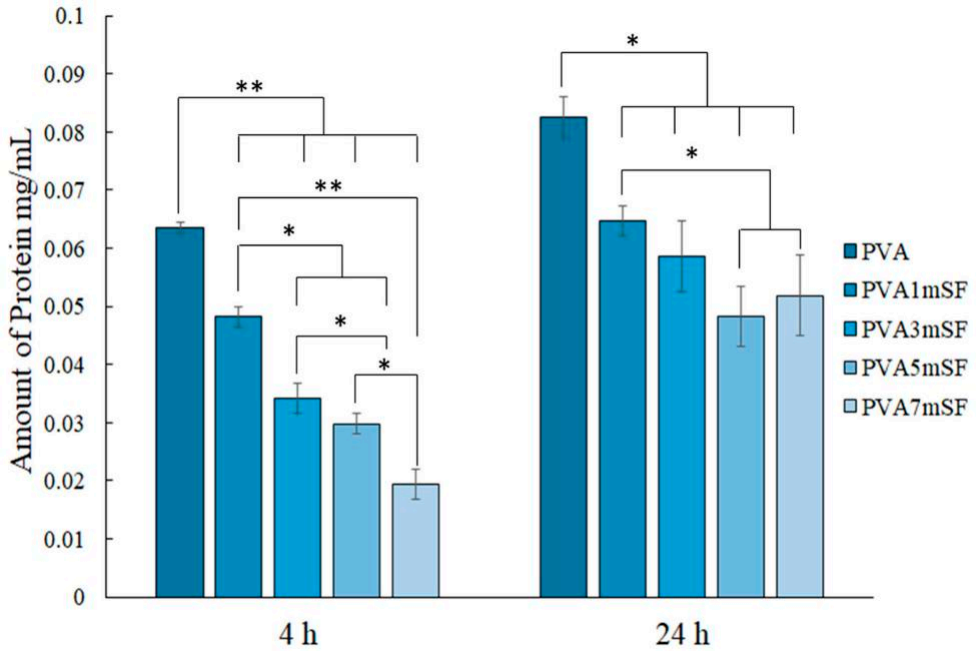
Protein adsorption of the mimic osteoid 3D porous scaffold in each group after 4 and 24 hours.

The protein adsorption observed at 4 and 24 hours on the scaffold indicates the initial interaction between the scaffold and the surrounding biological environment, which is an essential element for cell adhesion and subsequent tissue integration. The findings indicated that the pure PVA group exhibited the greatest protein adsorption during the initial 4 hours compared to the other groups. The inclusion of mSF significantly impacted the effectiveness of protein adsorption, particularly in the PVA7mSF group, which showed limited effectiveness. Protein adsorption positively correlated with the duration of time; all groups demonstrated an increase after 24 hours (Figure 8). There were no significant differences among PVA3mSF, PVA5mSF and PVA7mSF. However, PVA1mSF exhibited a significantly greater value compared to PVA5mSF and PVA7mSF. The pure PVA group exhibited significantly greater protein adsorption compared to the other groups.

### Cell adhesion assay

**Figure 9.**
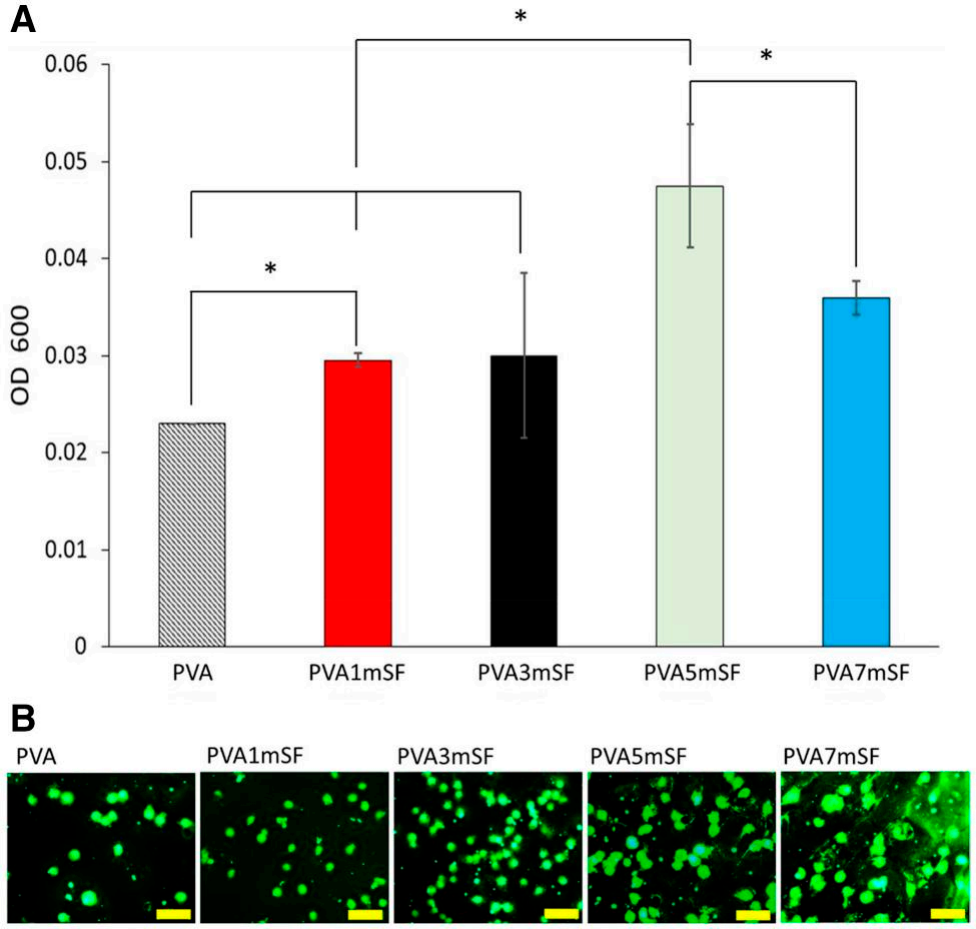
Cell adhesion efficacy on the mimic osteoid 3D porous scaffold in each group after 4 hours of cell seeding (**A**), and cell staining with FDA (**B**). The scale bar represents 100 µm.

The PVA5mSF mimic osteoid 3D porous scaffold exhibited superior cell adhesion compared to the other groups (Figure 9A and B). Conversely, the pure PVA mimic osteoid 3D porous scaffold showed reduced effectiveness in promoting cell attachment. Equivalent cell adhesion efficacy was observed in the PVA1mSF, PVA3mSF and PVA7mSF.

### Cell proliferation

**Figure 10.**
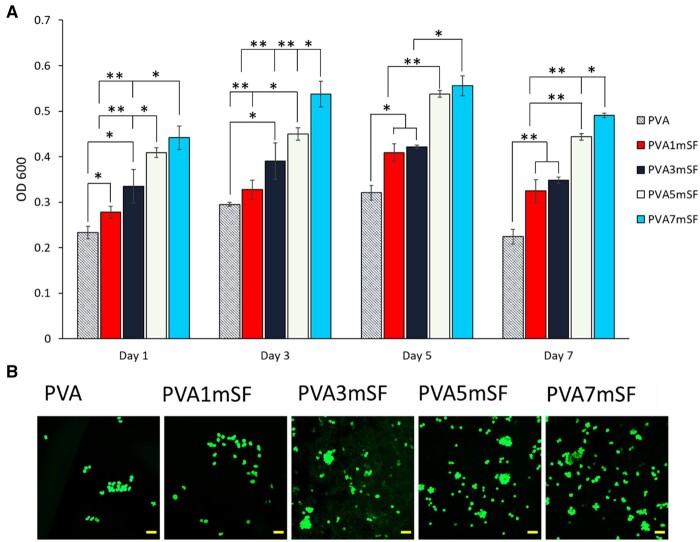
Cell proliferation of the mimic osteoid 3D porous scaffold in each group tested with PrestoBlue on days 1, 3, 5 and 7 (**A**) cell viability after day 1 (**B**). The scale bar represents 200 µm.

The cell proliferation trend exhibited an increase from day 1 to day 5, followed by a slight reduction after day 7 (Figure 10A and B). On day 1, the pure PVA mimic osteoid 3D porous scaffold exhibited significantly lower cell proliferation compared to the other groups. Significant cell proliferation was seen with increasing concentrations of mSF, namely at 1mSF, 3mSF, 5mSF and 7mSF, respectively. Significant cell proliferation was observed at higher concentrations of mSF, particularly in the PVA7mSF mimic osteoid 3D porous scaffold, followed by the PVA5mSF group on day 3. On day 5, there was a significant increase in cell proliferation for the PVA1mSF and PVA5mSF groups compared to the other groups. However, the PVA7mSF and PVA5mSF mimic osteoid 3D porous scaffolds exhibited similar levels of cell proliferation without any significant difference. Cell proliferation decreased in all groups of mimic osteoid 3D porous scaffold by day 7, with the pure PVA group showing significantly decreased proliferation compared to the PVA7mSF, PVA5mSF, PVA3mSF and PVA1mSF groups.

### Total protein assay

**Figure 11.**
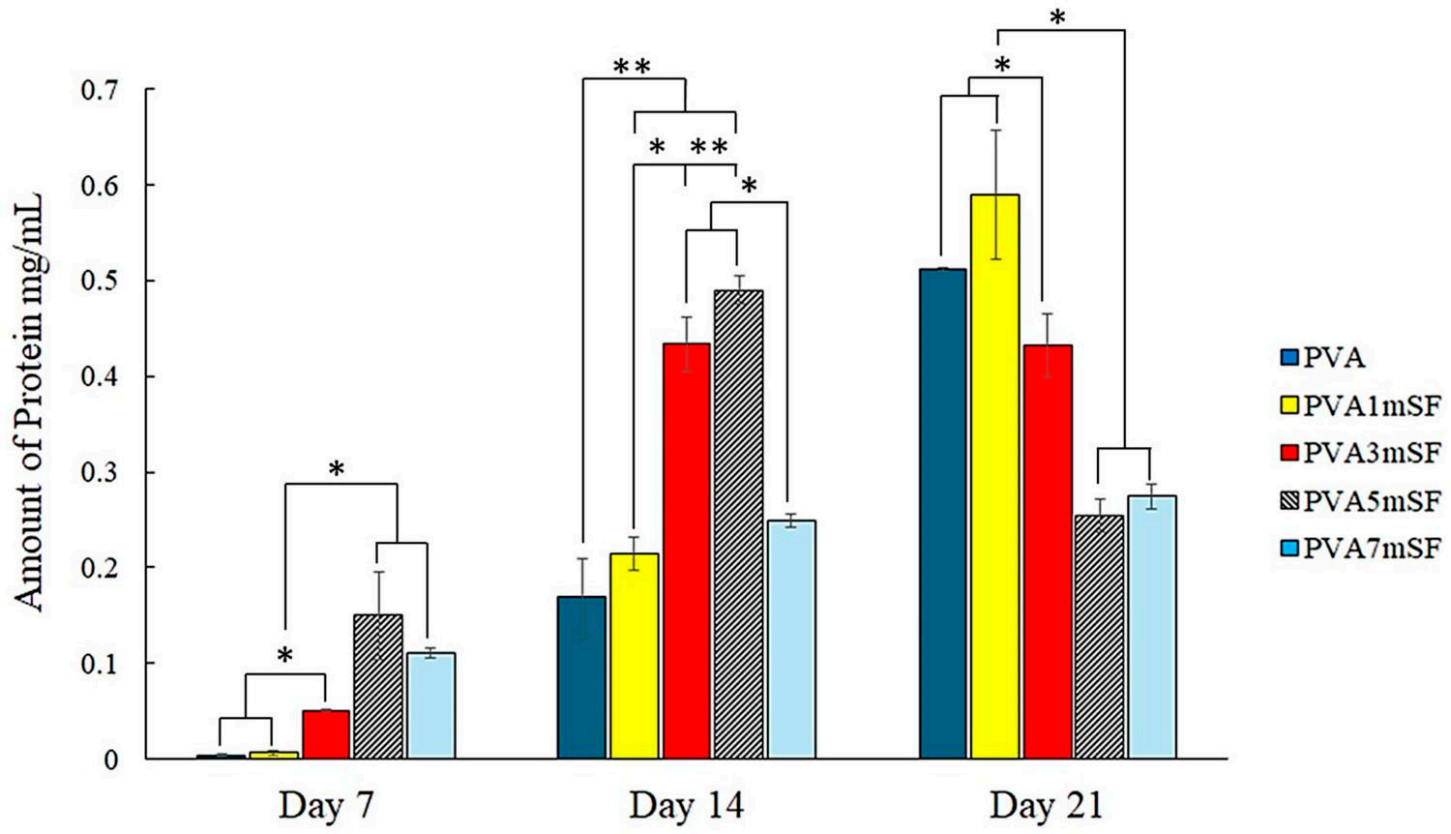
The protein synthesis of osteoblast cells after days 7, 14 and 21.

The protein synthesis and deposition matrix on the mimic osteoid 3D porous scaffold by osteoblast cells were measured on days 7, 14 and 21. The results showed that osteoblast cells exhibited increased protein synthesis on the modified PVA mimic osteoid 3D porous scaffold with a higher proportion of mSF (Figure 11). Specifically, the PVA5mSF group demonstrated the highest protein synthesis, followed by the PVA7mSF, PVA3mSF, PVA1mSF and PVA groups, respectively, on day 7. On day 14, an increase was observed in the PVA5mSF and PVA3mSF groups. However, after day 21, the protein synthesis of osteoblasts in the PVA osteoid 3D porous scaffold modified with mSF decreased in all groups. The PVA and PVA1mSF groups showed significant increases compared to the other groups.

### ALP activity

**Figure 12.**
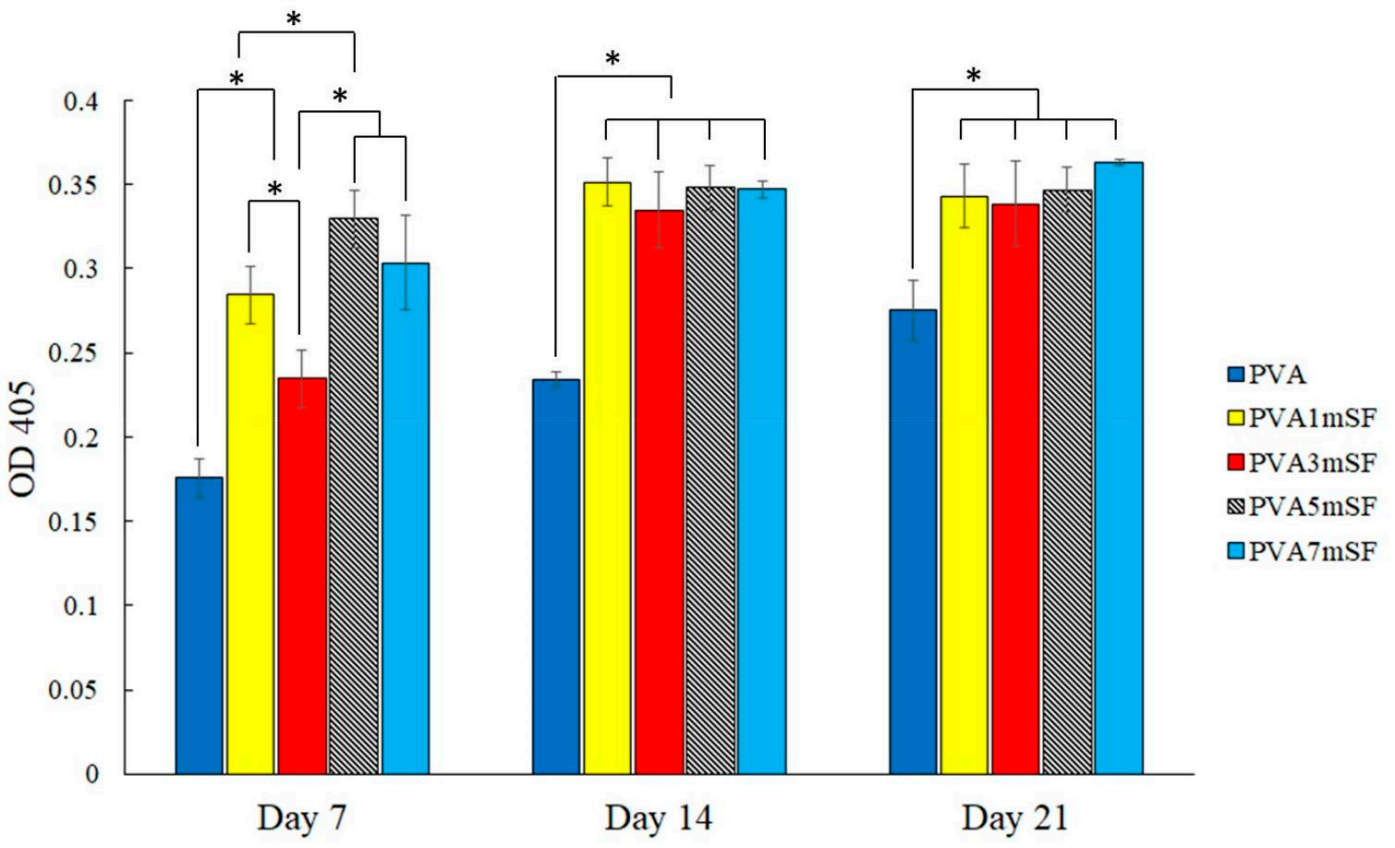
Alkaline phosphatase activity of osteoblast after days 7, 14 and 21.

ALP activity was assessed on days 7, 14 and 21 (Figure 12). The results indicated that enhanced ALP activity was achieved by modifying the 3D porous scaffold with mSF. Specifically, the ALP activity of PVA5mSF was considerably greater than that of PVA1mSF, PVA3mSF and PVA groups on day 7. ALP activity showed a slight increase after day 14, with no significant difference observed among the groups such as PVA1mSF, PVA3mSF, PVA5mSF and PVA7mSF. ALP activity declined after day 21 for PVA1mSF and PVA5mSF, whereas it increased in the PVA, PVA3mSF and PVA7mSF groups.

### Calcium synthesis

**Figure 13.**
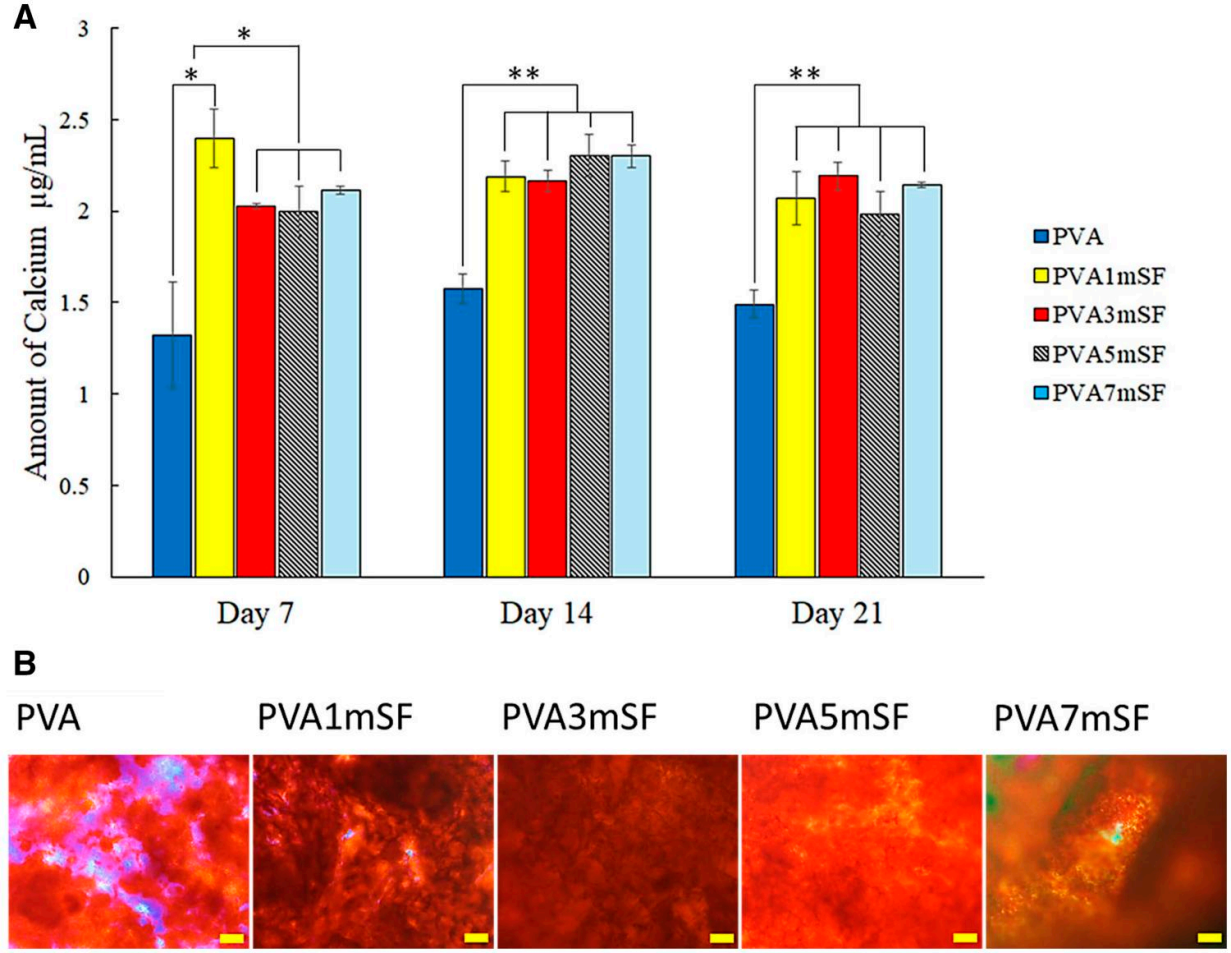
Calcium synthesis of osteoblast cell after days 7, 14 and 21 (**A**), calcium nodules deposition on the surface of the mimic osteoid 3D porous scaffold on day 14 (**B**). The scale bar represents 100 µm.

The calcium synthesis performance of osteoblast was evaluated after 7, 14 and 21 (Figure 13A). Alizarin red staining revealed calcium nodule deposition on day 21 by alizarin red staining (Figure 13B).

The PVA1mSF group exhibited significantly higher calcium synthesis compared to the other groups, particularly the PVA group. However, calcium synthesis in the PVA1mSF was decreased after day 14, and there was no significant difference among the PVA3mSF, PVA5mSF and PVA7mSF groups, although these groups showed significantly higher calcium synthesis compared to the PVA group. In contrast, calcium synthesis slightly decreased in all groups except the PVA3mSF group on day 21.

### Mimicking bone environment with dynamic condition

**Figure 14.**
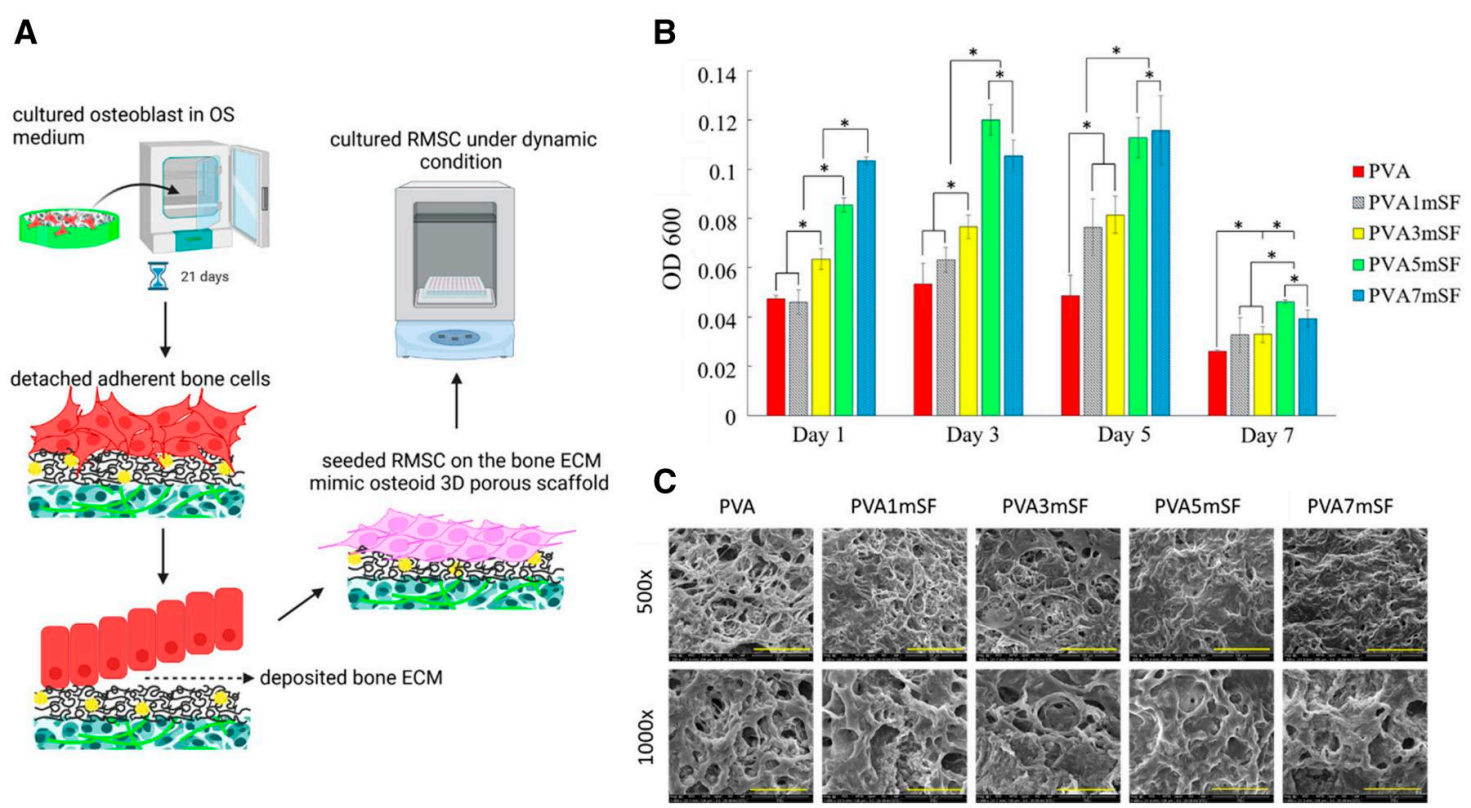
Schematic illustration of mimicking bone environment with dynamic condition (**A**), RMSC proliferation on the dECM osteoid 3D porous bone after days 1, 3, 5 and 7 (**B**), and SEM analysis of RMSC cell attachment after day 14 on the surface of the dECM osteoid 3D porous bone in each group (**C**). The scale bar represents 100 µm and 50 µm for ×500 and ×1000, respectively.

The results of cell proliferation showed that the modified mimic osteoid 3D porous PVA scaffold with a higher concentration of mSF significantly enhanced cell proliferation, particularly on days 1, 3 and 5, excluding day 7 (Figure 14B). On the first day, PVA7mSF exhibited a considerably greater value compared to all other groups, namely PVA5mSF, PVA3mSF, PVA1mSF and PVA. PVA5mSF exhibited a significant and accelerated augmentation in cell proliferation compared to PVA7mSF, surpassing the levels observed in the PVA3mSF, PVA1mSF and PVA groups. On day 5, there was a decrease in the trend of PVA and PVA5mSF, except for the PVA1mSF, PVA3mSF and PVA7mSF groups. The cell morphology and attachment were examined using an SEM analysis, and the results demonstrated that all groups exhibited favorable cell adhesion and cell migration. Notably, the PVA5mSF and PVA7mSF groups displayed significantly higher levels of cell adhesion and migration compared to the PVA, PVA1mSF and PVA3mSF groups (Figure 14C). PVA5mSF and PVA7mSF exhibited robust cellular proliferation, forming a continuous layer across the surface of the dECM osteoid 3D porous scaffold. Additionally, mineralization nodules were observed throughout the surface.

**Figure 15.**
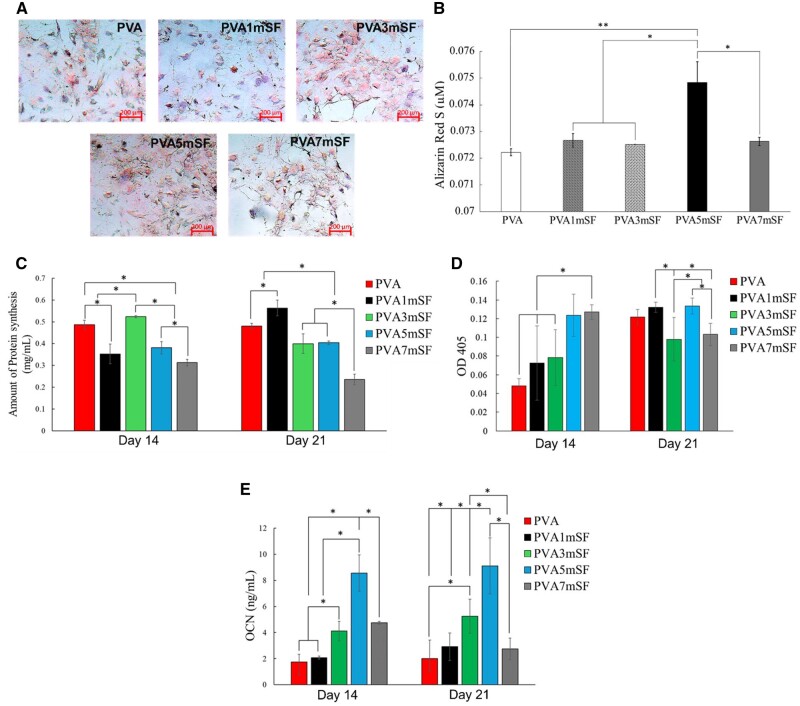
Performance differentiation of RMSC after cultured in dECM on day 14 under mimicking bone environment with dynamic condition experiment. Staining for calcium deposition (**A**), quantitative analysis of calcium deposition (**B**), protein synthesis (**C**), ALP activity (**D**) and OCN analysis (**E**).

The protein synthesis was assessed on days 14 and 21 (Figure 15C). The findings indicated that the PVA3mSF and PVA groups exhibited more protein synthesis compared to the PVA5mSF, PVA1mSF and PVA7mSF groups on day 14. On the 21st day, there was a notable increase in protein synthesis in the PVA1mSF group, which was considerably higher than in the other groups, namely PVA, PVA5mSF, PVA3mSF and PVA7mSF. On day 14, the higher proportion of mSF enhanced the ALP activity of RMSC, with the PVA7mSF group showing the highest ALP activity, followed by PVA5mSF, PVAPVA3mSF, PVA1mSF and PVA. On day 21, both the PVA and PVA1mSF groups showed a significant rise in ALP activity compared to day 14.

To evaluate the effectiveness of the dECM osteoid 3D porous scaffold in promoting the differentiation of RMSC into bone cells, the RMSC was co-cultivated with the dECM osteoid 3D porous scaffold while being rotated. The results demonstrated that all groups of RMSC were capable of producing calcium nodules, which appeared red after being stained with alizarin red on day 14 (Figure 15A). Quantitative analysis using alizarin red indicated that the PVA5mSF group had a considerably greater value compared to the other groups (Figure 15B). The OCN values obtained at 14 and 21 days serve as markers of the development of osteogenic differentiation and mineralization over a period of time. These values are crucial in assessing the efficacy of the scaffold in promoting bone regeneration. In addition, higher OCN levels, similar to the ALP result at the early stage on day 14, were observed, especially in the PVA osteoid 3D porous scaffold modified with higher proportions of mSF, including PVA5mSF, PVA7mSF, PVA3mSF and PVA1mSF, respectively. On day 21, PVA1mSF and PVA3mSF showed a clear increase in OCN levels compared to the other groups, except PVA7mSF, which decreased on this day (Figure 15D and E).

## Discussion

### Osteoid and calcification process in bone regeneration

Bone, a complex tissue, is composed of bioactive cells and a dense matrix of calcium phosphate and osteoid framework. Bone regeneration occurs through inflammation, soft callus formation, hard callus formation and remodeling. Osteoid and mineral deposition play crucial roles in bone formation, with osteoid forming the uncalcified matrix framework and serving as an indicator for mineral deposition [31]. Some literature suggests that the degree of osteoid formation and structural arrangement significantly influence the calcification and remodeling processes [32]. In bone tissue engineering, it is imperative and advantageous to develop materials that mimic the specific type of tissue defect, employing a concept that replicates biological procedures [33]. Such an approach greatly facilitates bone restoration. Our study utilized a mimic method to construct a 3D porous scaffold for ARP application, incorporating mSF as the fibrous protein embedded in PVA materials to mimic the glycosaminoglycan ground substance. Several reports indicated that increased osteoid synthesis facilitates calcification by enhancing the storage and deposition of calcium phosphate into the dense bone structure [34]. Furthermore, the effectiveness of this microenvironment regulates the behavior of MSCs in bone cells and influences the natural process of bone generation [35].

### Morphological structure, characterization and physical properties of mSF embedded PVA osteoid 3D porous scaffold for ARP

The study involved creating a mimic osteoid 3D porous scaffold containing mSF-embedded PVA. The sponge was chosen for this study due to its porous structure, which provides flexibility and facilitates cutting and trimming to adapt to the size of the defects [36, 37]. The osteoid 3D porous scaffold was designed to mimic the structure of the bone ECM, consisting of fibrous proteins within the GAG substance, as demonstrated by our study fabricating mSF within the interconnected pores of PVA. The fabrication of mSF followed the methodology described in Wonchan Lee et al.'s 2020 work. The mSF elements had a uniform size ranging from 50 to 150 µm, with an average size of 102.25 µm, enhancing their osteogenic functionality [38]. The fiber morphology of mSF formed a network of fibers embedded in PVA, resulting in a denser structure with smaller pore sizes due to the increased mSF concentration. The remaining mSF in the PVA enhanced the interconnected network structure of the osteoid 3D porous scaffold. This structure closely resembles the dense structure of the bone ECM, which is formed by the arrangement of collagen fibers in the compact layer for mineral deposition [39]. The crystalline structure is a significant characteristic of the material, closely linked to the mechanical properties of the osteoid 3D porous scaffold [40, 41]. Several studies have indicated that the presence of a crystalline structure in the osteoid 3D porous scaffold enhances its mechanical characteristics and increases the area available for cell attachment, as opposed to amorphous structures [42]. Our mimic osteoid 3D porous scaffold exhibited a prominent peak at 2θ = 19.49˚ to 19.59˚, attributed to the mSF fiber that was not fully dissolved in sodium hydroxide [43]. The major and minor peaks observed at 19.46˚ and 40.63˚ correspond to the PVA crystalline structure [44]. The arrangement of these structures, comprising both crystalline and amorphous structures, closely resembles the organization found in the ECM of bones [45]. Our findings demonstrated a positive correlation between the percentage of mSF and the melting point of the mimic osteoid 3D porous scaffold. Multiple studies indicate that microfiber SF has a higher melting point compared to PVA and enhances the thermal responsiveness of the PVA osteoid 3D porous scaffold [46]. The wettability property of the pure PVA osteoid 3D porous scaffold exhibited superior hydrophilicity compared to other groups, especially in the higher mSF portion, due to the inherent hydrophilic properties of the PVA and SF polymers [47]. Additionally, the SF polymer contains hydrophobic regions composed of alanine and glycine motif peptides, which are arranged in a β-sheet shape [48]. Several studies have reported using microfiber SF as a reinforcement material [49]. These results indicate that incorporating microfiber SF into PVA enhances mechanical properties, including maximum loading, Young’s modulus and stiffness. Furthermore, the swelling analysis revealed that the incorporation of mSF into PVA resulted in an increased swelling percentage of the PVA osteoid 3D porous scaffold. This can be attributed to the interconnected pore structure created by mSF within the PVA material [50]. Several studies indicate that the interconnected pore size and increased porosity of the osteoid 3D porous scaffold provide a larger area for water retention [51]. However, osteoid 3D porous scaffolds with larger porosity have several disadvantages [52]. The homogenous texture of pure PVA contributed to its ability to preserve the shape and structure of the osteoid 3D porous scaffold. In contrast, the higher mSF groups, such as PVA5mSF and PVA1mSF, exhibited a lower degradation rate compared to the pure PVA group. The lower surface area of PVA made it more susceptible to degradation, while SF, with its richer β-sheet structure, was more resistant to degradation than PVA [53].

### Biofunctional of osteoblast cell response to an mSF-embedded PVA osteoid 3D porous scaffold

Evaluating the biocompatibility and cell response behavior of the mimic osteoid 3D porous scaffold is crucial for assessing its biofunctionality. Our study found that the PVA osteoid 3D porous scaffold, when combined with mSF, significantly enhanced cell adhesion, especially in the PVA5mSF group. The initial 4-hour period is critical for cell attachment, as it facilitates cell communication and regulates new tissue regeneration [54]. Cell adhesion performance is influenced by various factors, such as surface roughness, rigidity, topography and the specific type of material, particularly protein polymers, which are preferred for cell attachment [55]. Multiple studies have demonstrated that fibrous proteins have strong cell adhesion capabilities through protein-specific regions such as arginyl, glycyl and aspartic acid (RGD) [56]. This peptide motif, known as RGD, facilitates cell adhesion. Microfiber SF contains RGD-integrin-binding peptides that enhance cell adherence during the initial phases [57]. Furthermore, the arrangement of fiber protein in the PVA polymer closely resembled the structure of the bone ECM, which consists of fibrous protein embedded in a substance called GAG [9, 58]. During the biocompatibility testing, the samples were kept in the incubator for a period of 7 days, from day 1 to day 7. This controlled environment effectively caused the mimic osteoid 3D porous bone scaffolds to degrade. Nevertheless, the significant amount of PVA that remains after degradation acts as a molecule that absorbs proteins, thereby promoting cell growth through its interaction with proteins from the medium or blood, which are capable of supporting cell growth [21]. The study investigated cell proliferation by evaluating the PVA osteoid 3D porous scaffold modified with various percentages of mSF between days 1 and 7. The results demonstrated that the modification significantly impacted cell proliferation. Adding different concentrations of mSF to the PVA resulted in enhanced cell proliferation from day 1 to day 7. This enhancement can be attributed to the stability of mSF, its appropriate pore size and interconnective structure, surface roughness and the presence of bioactive features such as RGD motif peptides.

### Mineralization performance of osteoblast cell on the mSF-embedded PVA osteoid 3D porous scaffold

Mineralization in bone regeneration involves osteoblast cells producing collagen and proteoglycans, secreting calcium and phosphate and promoting new bone growth by regulating RMSC in bone cells and synthesizing minerals [59]. This study fabricated a PVA osteoid 3D porous scaffold with varying amounts of mSF as an additive. Protein synthesis increased during differentiation stages, particularly between days 7 and 14, with the PVA5mSF and PVA3mSF groups showing high levels. Protein synthesis decreased in a higher proportion of mSF groups. This occurrence was associated with the stage of bone regeneration [60]. Between days 7 and 14, protein synthesis increased to create an organic matrix for mineral deposition. Subsequently, protein synthesis gradually decreased, giving way to calcium phosphate synthesis [61]. Additional bone markers assessed in this investigation included ALP activity and calcium production. The findings demonstrated that the addition of mSF resulted in enhanced mineral synthesis compared to the PVA group. Extensive research has shown that the mineral synthesis of osteoblast cells during development in the mimic osteoid 3D porous scaffold is regulated by several crucial variables. Fiber-protein-based materials are preferred by osteoblast cells due to the presence of bioactive chemicals in the protein polymer and their ability to mimic the structure and components of the bone ECM [62]. Additionally, the porous material exhibits an interconnective pattern that closely resembles the structure of bone [63]. The stability and rigidity of the osteoid 3D porous scaffold contribute to long-term cell adhesion and proliferation [42, 64]. Furthermore, the remaining SF fiber allowed more effective mineral deposition than the pure PVA polymer. However, the highly hydrophilic nature of PVA hindered cell attachment because it significantly increased the surface area of the soft osteoid 3D porous scaffold over an extended period [65].

### The ability of mSF-embedded PVA osteoid 3D porous scaffold to promote bone growth through osteoconductive characteristics

The osteoconductive activity of the osteoid 3D porous scaffold was assessed using a model that mimics a bone environment under dynamic conditions. We developed a bone environment with a dynamic condition model using a dECM mimic osteoid 3D porous scaffold and cultivated it with RMSC in a dynamic environment without the need for osteogenic supplement reagent media. Several studies discuss the use of dynamic conditions in cell culture, which can mimic the mobility of bones [66]. The dECM represents the secretion of the bone matrix during the mineralization phase. Our objective was to evaluate the efficacy of dECM in different groups of mimic osteoid 3D porous scaffolds, focusing on its ability to enhance RMSC growth and facilitate their transformation into bone cells. The study examined cell proliferation, protein synthesis, ALP activity and OCN levels and performed an indirect test to observe cell responses to the mimic osteoid 3D porous scaffold during the implantation procedure. Compared to other groups, RMSC proliferation was significantly higher in the high percentage of SF microfiber dECM groups, particularly in PVA5mSF and PVA7mSF, as well as the increased matrix deposition from osteoblasts. All groups of RMSC cells demonstrated a positive reaction to the dECM mimics osteoid 3D porous scaffolds during the indirect test, particularly under dynamic conditions. The PVA5mSF group showed a greater alizarin red quantitative analysis after day 14 compared to the other groups, whereas the PVA group demonstrated lower results. The PVA and PVA osteoid 3D porous scaffold additives, specifically PVA1mSF and PVA3mSF, with a smaller portion of mSF, exhibited higher levels of protein synthesis. This phenomenon occurred due to increased mineral deposition in the dECM of the PVA5mSF and PVA7mSF groups. These groups have a greater ability to regulate the differentiation stage of RMSC and are particularly effective in synthesizing the mineral matrix compared to other groups. The bone marker study included measurements of ALP activity and OCN levels. The findings revealed that the PVA5mSF group exhibited elevated ALP activity on days 14 and 21. Furthermore, PVA5mSF showed an outstanding OCN value on both days 14 and 21, attributed to the optimal ratio between SF microfiber and PVA. This microenvironment closely mimics the ECM of bones, which contains substances that facilitate protein adsorption. Additionally, the ECM contains fiber proteins embedded with calcium and phosphate, secreted by osteoblasts. This combination creates a conducive environment for the controlled growth and differentiation of RMSC in bone regeneration [67].

## Conclusion

The study successfully developed a mimic osteoid 3D porous scaffold embedded with mSF and PVA components for alveolar ridge preservation. The addition of mSF improved both the scaffold’s porous architecture and mechanical properties, with higher mSF percentages indicating superior biological performance. The PVA5mSF group exhibited improved bone matrix synthesis and deposition. In a dynamic environment, the dECM PVA5mSF scaffold further promoted RMSC proliferation, ALP activity and OCN synthesis, indicating successful modulation of bone cell function.
